# Supplementary material for: Graphene-driven correlated electronic states in one dimensional defects within WS2
Source: Nat Commun. 2025 Jul 1;16:5809. doi: 10.1038/s41467-025-60993-x (PMC12215652; doi:10.1038/s41467-025-60993-x)
Supplement: Supplementary file 1 — Supplementary Information [file 41467_2025_60993_MOESM1_ESM.pdf]

Supplementary Information for Graphene-Driven Correlated Electronic States in One Dimensional Defects within WS_2

Antonio Rossi[1,2,3*†], John C. Thomas[1,3*†], Johannes T. Küchle[1,4], Elyse Barré[1], Zhuohang Yu[5,6], Da Zhou[7], Shalini Kumari[5,6], Hsin-Zon Tsai[8], Ed Wong[1], Chris Jozwiak[2], Aaron Bostwick[2], Joshua A. Robinson[5,6,7,9], Mauricio Terrones[5,6,7,9], Archana Raja[1], Adam Schwartzberg[1], D. Frank Ogletree[1], Jeffrey B. Neaton[3,8,10], Michael F. Crommie[3,8,10], Francesco Allegretti[4], Willi Auwärter[4], Eli Rotenberg[2], and Alexander Weber-Bargioni[1,3*]

[1]*Molecular Foundry, Lawrence Berkeley National Laboratory, Berkeley, CA, 94720, United States of America*
[2]*Advanced Light Source, Lawrence Berkeley National Laboratory, Berkeley, CA, 94720, United States of America*
[3]*Materials Sciences Division, Lawrence Berkeley National Laboratory, Berkeley, CA, 94720, United States of America*
[4]*Physics Department E20, TUM School of Natural Sciences, Technical University of Munich, D-85748, Garching, Germany*
[5]*Department of Materials Science and Engineering, The Pennsylvania State University, University Park, PA, 16082 United States of America*
[6]*Center for Two-Dimensional and Layered Materials, The Pennsylvania State University, University Park, PA, 16802 United States of America*
[7]*Department of Physics, The Pennsylvania State University, University Park, PA, 16802 United States of America*
[8]*Department of Physics, University of California at Berkeley, Berkeley, CA, 94720, United States of America*
[9]*Department of Chemistry, The Pennsylvania State University, University Park, PA, 16802 United States of America*
[10]*Kavli Energy NanoSciences Institute, University of California Berkeley, Berkeley, CA, 94720, United States of America*
[*]*jthomas@lbl.gov, arossi@lbl.gov, afweber-bargioni@lbl.gov*
[†]*These authors contributed equally.*

Supplementary Notes

1| Ar^+ sputtering and SRIM simulations

Monte Carlo simulations based on The Stopping and Range of Ions in Matter, SRIM simulations[1], were used to evaluate preparation conditions using Ar^+ bombardment. The Transport of Ions in Matter (TRIM) calculation, which assumes amorphous targets, with 50,000 ions was determined to be sufficient for simulation convergence; between simulating 20,000 and 50,000 ions, the variation in the number of vacancies created was less than 2% for all possible atomic vacancies. The Ar^+ energy was set to 0.1 keV to gauge angle dependence and fixed at an angle of 60° to gauge energy dependence, where these values were chosen to be used experimentally. We set the height of WS_2 to 0.72 nm, the height of graphene to

0.34 nm, and the height of SiC to 30 nm[2,3]. Density, displacement energy, and surface binding energy values were matched to literature values[4–6] .

Using the estimated argon flux of $1.5\times10^{13}\ \frac{ions}{cm^2 s}$ at 30 seconds of irradiation, we obtain a value of 4.5 $\frac{ions}{nm^2}$. Each ion is predicted to induce 3 V_S, or 13.5 $\frac{V_S}{nm^2}$. Considering the local measurement of $0.168 \pm 0.052\ \frac{V_S}{nm^2}$ and 1D metal (1DM) formation of $0.021 \pm 0.009\ \frac{1DM}{nm^2}$ with a length of 3.37 ± 2.87 nm. We use simulation results to estimate defect creation and local measurements to approximate defect density.

2| Defect analysis

An object class can be instantiated within Python, where each image can be loaded for analysis with a given size (nm). Defects are selected initially by inspection, and local minima or maxima are calculated within a given pixel range. Each selected defect is then cross-checked and input into a graph, where density can be calculated by the number of defects within a the given area, assuming an equal N×N image.

```
class node(object):
    def __init__(self,position,value):
        self.value=value
        self.position=position
    def getPosition(self):
        return self.position
    def getvalue(self):
        return self.value
    def getNodeHash(self):
        return hash(str(self.position)+str(self.value))
    def __str__(self):
        return str('Pos:'+str(self.position)+' Val:'+str(self.value))

class edge(object):
    def __init__(self,src,dest):
        self.src = src
        self.dest = dest
    def getSource(self):
        return self.src
    def getDestination(self):
        return self.dest
    def getWeight(self):
        return self.dest.getvalue()
    def __str__(self):
        return str(self.src.getPosition())+'-->'+str(self.dest.getPosition())

class emap(object):
    def __init__(self):
        self.edges = {}
    def addNode(self,node):
        if node in self.edges:
            raise ValueError('Duplicate node')
        else:
            self.edges[node]=[]
    def addEdge(self,edge):
        src = edge.getSource()
        dest = edge.getDestination()
```

```
        if not (src in self.edges and dest in self.edges):
            raise ValueError('Node not in graph')
        self.edges[src].append(dest)
    def getChildrenof(self,node):
        return self.edges[node]
    def hasNode(self,node):
        return node in self.edges
    def display(self):
        for i in self.edges:
            print(i)
    def getedgelen(self):
        return len(self.edges)

class defect_map(object):
    def __init__(self):
        self.len=0
        self.images = []
        self.imsize = []
        self.density = []
    def addimage(self,im,siz):
        self.images.append(im)
        self.imsize.append(siz)
        self.len += 1
    def getlen(self):
        return self.len
    def select_defects(self, win):
        for idx in range(0,len(self.images)):
            outpts = selectdefects(self.images[idx],win,idx)
            dList = emap()
            nlist = []
            k = 0
            for x, y, z in outpts:
                mol=node([x,y],k)
                dList.addNode(mol)
                nlist.append(mol)
                k += 1
            visited = []
            for i in nlist:
                visited.append(i)
                for j in nlist:
                    if j not in visited:
                        dList.addEdge(edge(i,j))
            self.density.append(dList.getedgelen())
    def getdensity(self):
        tmp = []
        for i in range(0,len(self.density)):
            tmp.append(self.density[i]/(self.imsize[i]**2))
        return tmp
```

3| Tomonaga Luttinger Liquid (TLL)

A TLL low-energy Hamiltonian in a box can be defined as[7–12]

$$H_{TLL} = \frac{\pi v_c N^2}{4LK_c} + \frac{\pi v_s S_s^2}{LK_s} + \sum_{n=1}^{\infty} (v_c k_n a^{\dagger}_{c,n} a_{c,n} + v_s k_n a^{\dagger}_{s,n} a_{s,n}),$$

where L is defect length, c and s label the charge and spin channels, K_c and K_s are two Luttinger parameters, v_c and v_s are charge and spin velocities, N is the total electron filling, S_z is the total z-component spin number, and $a^{\dagger}_{c,n}$ and $a^{\dagger}_{s,n}$ are the creation operators of charge and spin excitation. This representation identifies the key requirements for a TLL to be present within an ID. These requirements are such that 1) the first and second terms define a charging energy that determines the HOS-LUS energy gap (E_{gap}) arising from Coulomb interactions and the spin sector, respectively, 2) spin and charge show independent dispersions, 3) there exists E_{gap} dependence that is inversely proportional to an ID length, and 4) creation operators give rise bosonic excitations of both spin and charge to yield a lateral and energetic dependence that exhibits particle-in-a-box behavior. In the results presented, all requirements are fulfilled to showcase the presence of a TLL hosted by an ID within WS_2.

SUPPLEMENTARY FIGURES

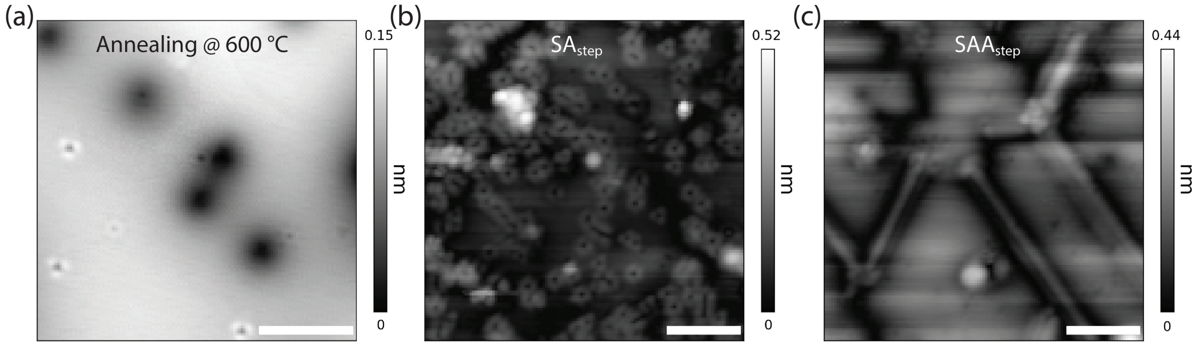

WS_2/EG-SiC(0001) Prep	1D (defects/nm^2)	Point Vacancies (defects/nm^2)
Anneal, 600 °C	0	0.010 +/- 0.002
SA$_{step}$: Ar^+ Bombardment, 600 °C	0.021 +/- 0.009	0.168 +/- 0.052
SAA$_{step}$: Ar^+ Bombardment, 600 °C + 30 min anneal, 600 °C	0.009 +/- 0.001	0.004 +/- 0.003

Supplementary Fig. 1: **Defect Density.** Defective density is calculated as the number of defects per unit area, where (a) annealed only samples show a low defect density, (b) sputtered with annealing produces a larger number of both 1D defects and V_S, and (c) sputtered with annealing plus an additional 30 minute anneal produces elongated 1D defects with less V_S (I_{tunnel} = 30 pA, V_{sample} = 1.2 V). Scale bars, 4 nm. Each defect, across a large number of images over multiple samples and subsequent preparations, is selected by inspection and then by solving for the local minima or maxima within a given pixel window.

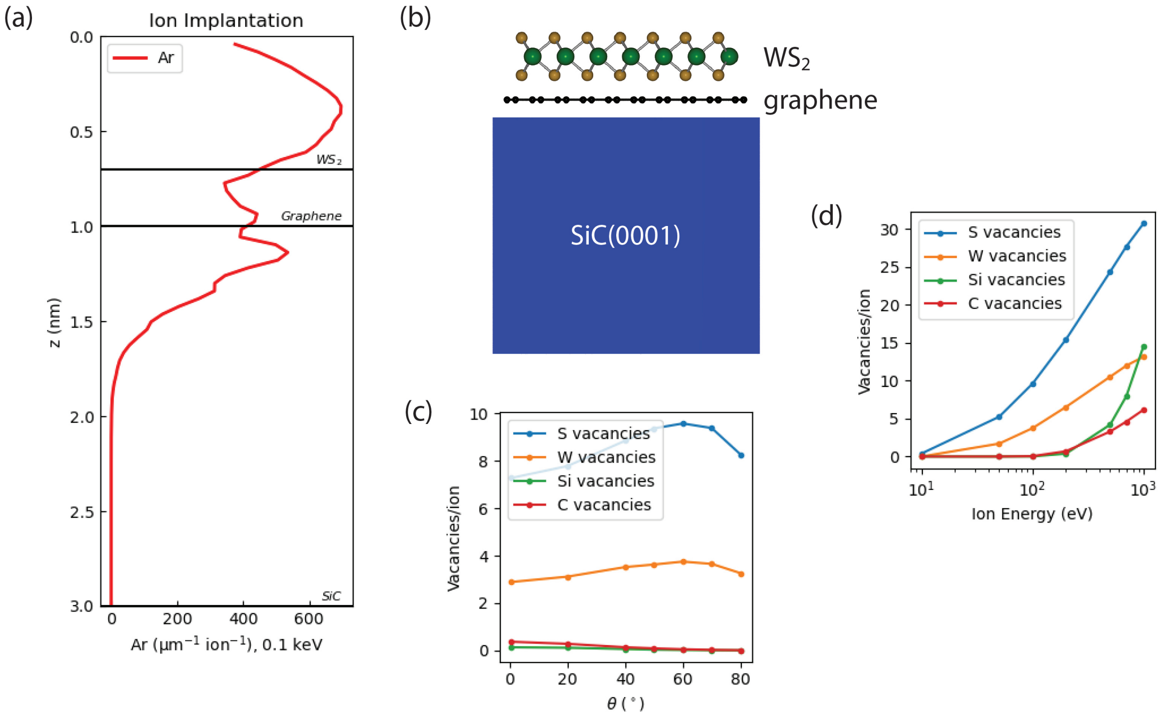

Supplementary Fig. 2: **SRIM Simulations.** (a) Results of SRIM simulations with 50000 ions for a (b) WS_2/Graphene/SiC(0001) heterostructure, where Ar^+ ions are expected to nominally interact with the TMD overlayer given the ion energy and angle of irradiation incidence. Both (c) and (d) depict the number of vacancies produced over a given incidence angle and energy, where we use an energy of 0.1 keV and an angle of 60°, respectively.

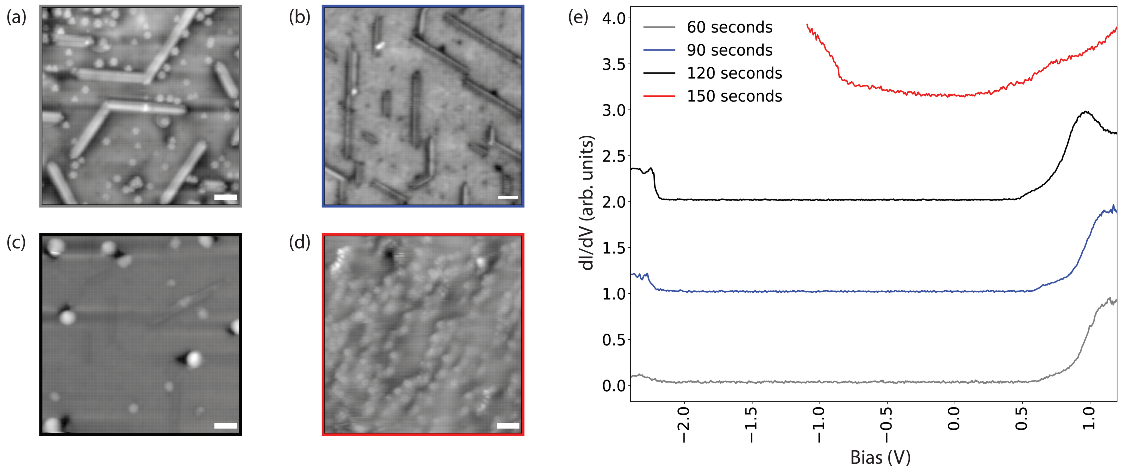

Supplementary Fig. 3: **1DM Elongation and WS_2 Degradation.** Constant current image over WS_2 after (a) two SAA_{step} (I_{tunnel} = 20 pA, V_{sample} = 1.2 V), (b) three SAA_{step} (I_{tunnel} = 20 pA, V_{sample} = 1.4 V), (c) four SAA_{step} (I_{tunnel} = 25 pA, V_{sample} = 1.6 V), and (d) five SAA_{step} (I_{tunnel} = 25 pA, V_{sample} = 1.6 V). Each cycle consists of a 30 second sputter and additional anneal. Scale bars, 4 nm. (e) dI/dV spectra recorded over non defective regions at each step and labeled by the total amount of sputter time ($V_{modulation}$ = 5 mV). The expected E_{gap} of WS_2 decreases above four SAA_{step} and gains a metallic character, which is associated with material degradation.

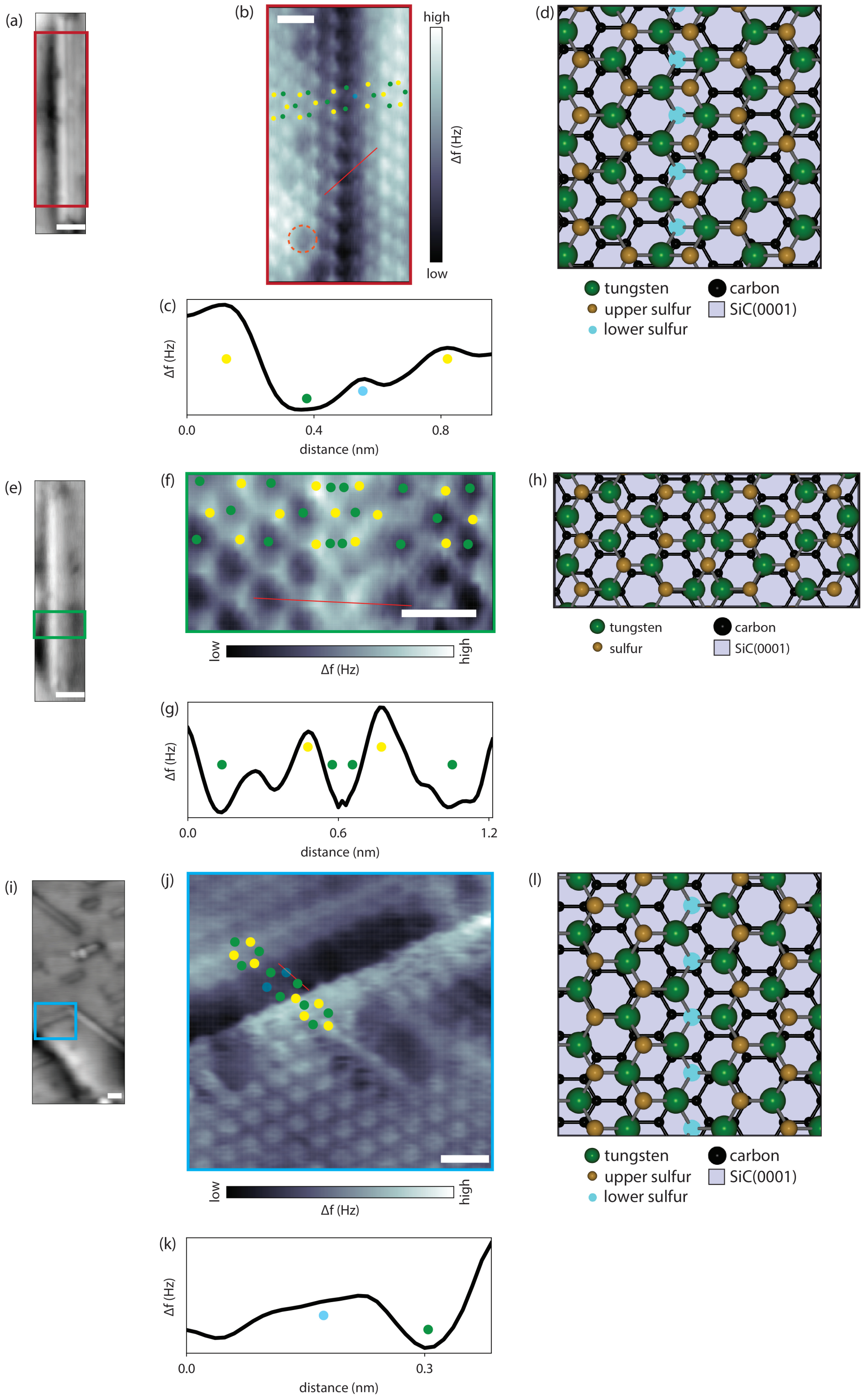

Supplementary Fig. 4: **Atomic Force Imaging.** (a) Constant current image over an isolated 4|4E intermediate 1DM defect (I_{tunnel} = 30 pA, V_{sample} = 1.2 V). Scale bar, 1.5 nm. (b) ncAFM (V_{sample} = 0.0 V) collected over the same 4|4E intermediate 1DM with a ball model showing atomic locations. Scale bar, 0.4 nm. (c) A linescan over the red region shown in (b) that depicts two types of sulfur measured, where one is assigned to the top position (yellow) and the other to the bottom position (cyan) where metal sites appear as dips in frequency shift images. Depressions near the 4|4E intermediate 1DM reflect oxygen atom (circled in orange) chalcogen substitutions within an otherwise unmodified WS_2 lattice. A structure model is shown schematically in (d). A second isolated 1DM is shown in (e) that is identified to be an 4|4P intermediate 1DM (I_{tunnel} = 30 pA, V_{sample} = 1.2 V). Scale bar, 1.5 nm. (f) ncAFM confirms the location of top sulfur, tungsten, and hollow sites (V_{sample} = 0.0 V), where a linescan of across the 4|4P intermediate 1DM in (g) shows the location of dual tungsten surrounded by sulfur sites that is also shown schematically in (h). Scale bar, 0.4 nm. A 1DM chalcogen vacancy line is shown in (i) (I_{tunnel} = 30 pA, V_{sample} = 1.2 V). Scale bar, 1.5 nm. (j) Sulfur vacancies are measured in ncAFM (V_{sample} = 0.0 V). A linescan across the 1DM in (k) shows the tungsten and lower sulfur sites, schematically depicted in (l). Scale bar, 0.4 nm.

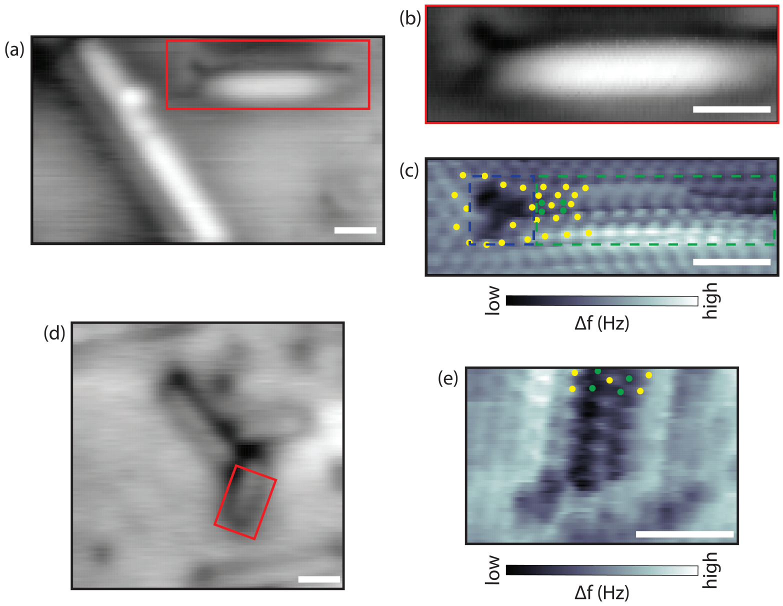

Supplementary Fig. 5: **1DM Defect End Point Mapping.** (a) Constant current image over an isolated 1DM defect (I_{tunnel} = 30 pA, V_{sample} = 1.2 V) terminating in an end point defect. Scale bar, 1 nm. (b) Zoomed in image that is highlighted in (a) with a red box, where sequential (c) ncAFM with a CO functionalized tip (V_{sample} = 0.0 V) collected over the same 1DM showcase local structure. Scale bars, 1 nm. A chalcogen depletion region (blue) is located at the edge of the isolated and strained 1DM(further highlighted in green). Chalcogen (sulfur) sites are highlighted with yellow spheres and metal (tungsten) sites are highlighted with green spheres. (d) Another defect is measured that appears as an intermediate formation before a fully relaxed MTB (I_{tunnel} = 30 pA, V_{sample} = 1.2 V). Scale bar, 1nm. ncAFM over an endpoint region (highlighted with a red box) shows strained metallic-rich regions that form into a 4|4P intermediate structure.

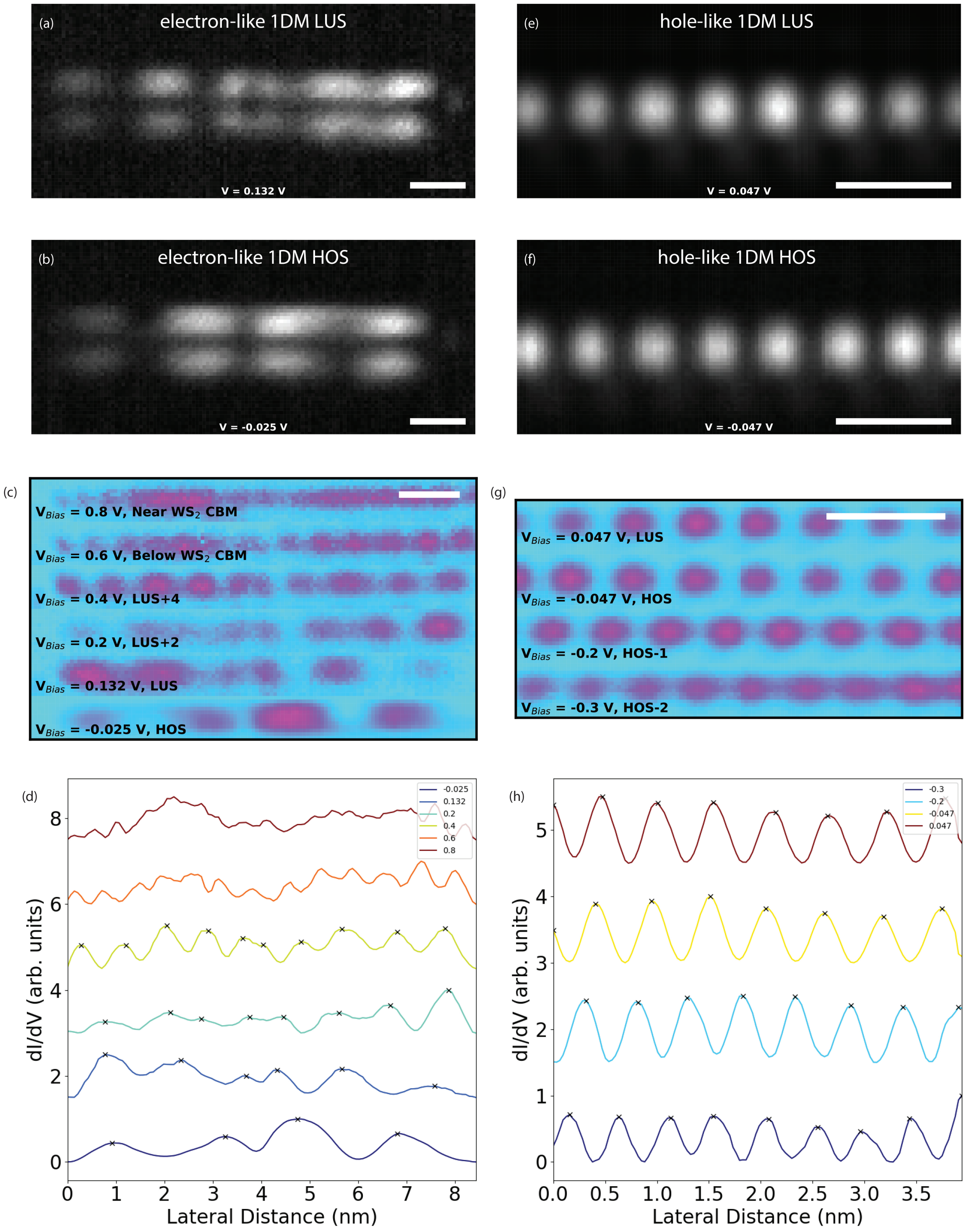

Supplementary Fig. 6: **LDOS Mapping.** Conductance maps ($V_{modulation}$ = 5 mV) performed across an electron-like 1DM show dual-line orbital behavior at (a) 0.132 eV (LUS) and (b) -0.025 eV (HOS) that is spatially out of phase. (c) Accumulated conductance maps across a single-line of the electron-like 1DM ($V_{modulation}$ = 5 mV) are further shown as a function of bias, where the number of nodes increase as bias voltage is increased. Scale bars, 1 nm. Peak assignments can be made by solving for local maxima along a line profile, which is compiled in (d). Conductance maps (dI/dV) of the as-measured (e) 0.047 eV (LUS) and (f) -0.047 eV (HOS) that are spatially in phase within an hole-like 1DM. (g) Compiled conductance maps ($V_{modulation}$ = 5 mV) across the single-line hole-like 1DM are further shown, where the number of nodes decreases as the voltage is increased. (h) Line profiles extracted from (g) detailing local maxima.

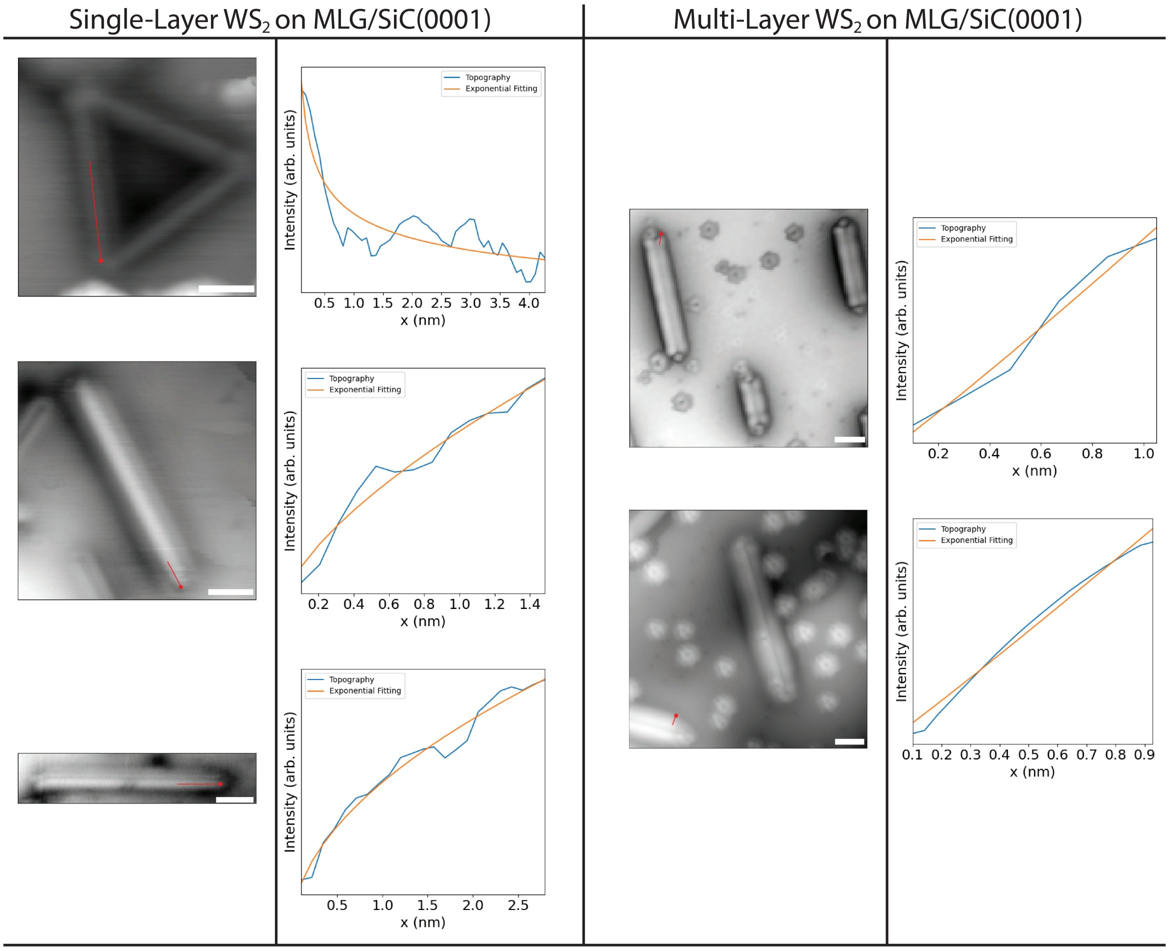

Supplementary Fig. 7: **Constant-Current Density of States Decay Across a 1DM.** A power-law dependence is measured across a multiple 1DM defects on both monolayer WS_2 and multilayer (3ML) WS_2 on MLG/SiC(0001). In each image depicted (V_{sample} = 1.2 V), multiple fits were taken across defects shown, where representative linescans beginning at a starred point and along a given line (red) are shown with corresponding constant-current profiles with subsequent exponential fittings. Across 14 lineprofiles and 10 defects, multilayer WS_2 defects show an absolute power-law exponential parameter of 0.99 ± 0.15, which indicates Fermionic behavior, and monolayer WS_2 defects yield a parameter of 0.49 ± 0.16 that matches expected behavior of a Luttinger liquid. Scale bars, 2nm. Fittings were performed using the lmfit package in Python [13].

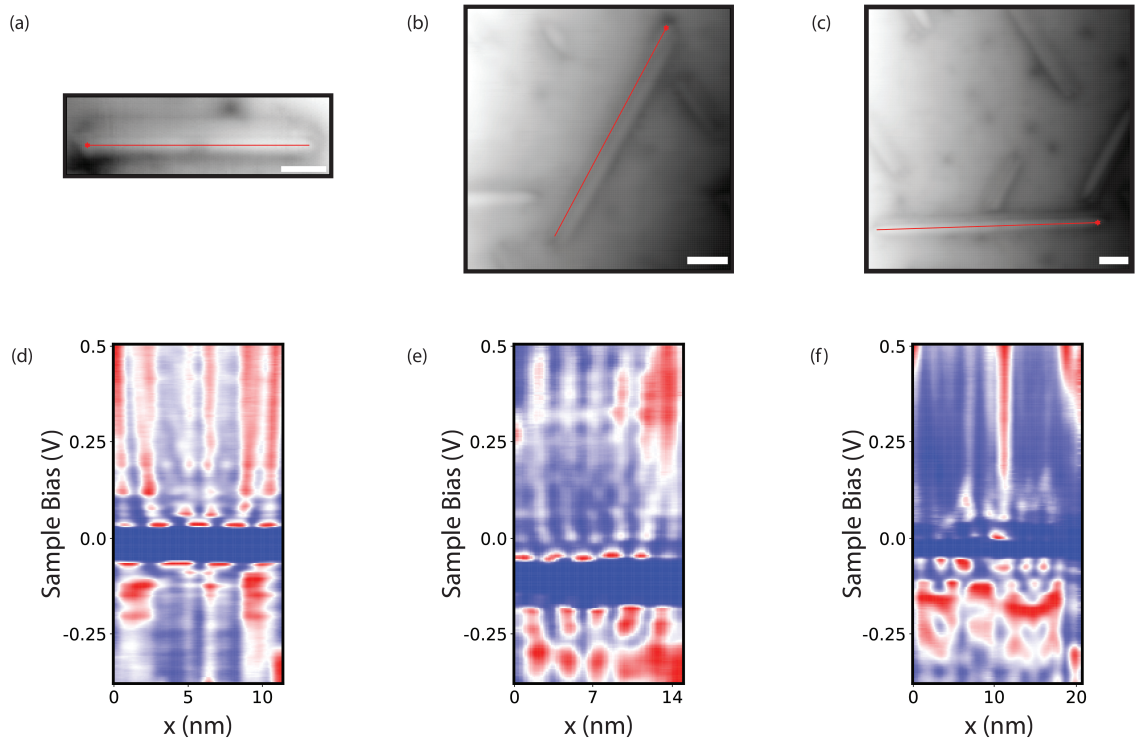

Supplementary Fig. 8: **Electron-like 1DM Dispersion Length Dependence.** Electron-like dispersions are collected on 1DMs with different lengths. Constant current images over 1DMs that are (a) 11.19 nm (I_{tunnel} = 30 pA, V_{sample} = 1.2 V), (b) 14.86 nm (I_{tunnel} = 30 pA, V_{sample} = 1.4 V), and (c) 20.44 nm (I_{tunnel} = 80 pA, V_{sample} = 1.2 V) are collected. Scale bars, 2 nm. Dense scanning tunneling spectra ($V_{modulation}$ = 5 mV, I_{set} = 150 pA) are collected along the red line, beginning at the starred point. Results are shown for the (d) 11.19 nm defect (1x128x500 pixels), (e) 14.86 nm defect (1x128x400 pixels), and the (f) 20.44 nm defect (1x128x400 pixels). Using the HOS as a reference, a node spacing of is measured.

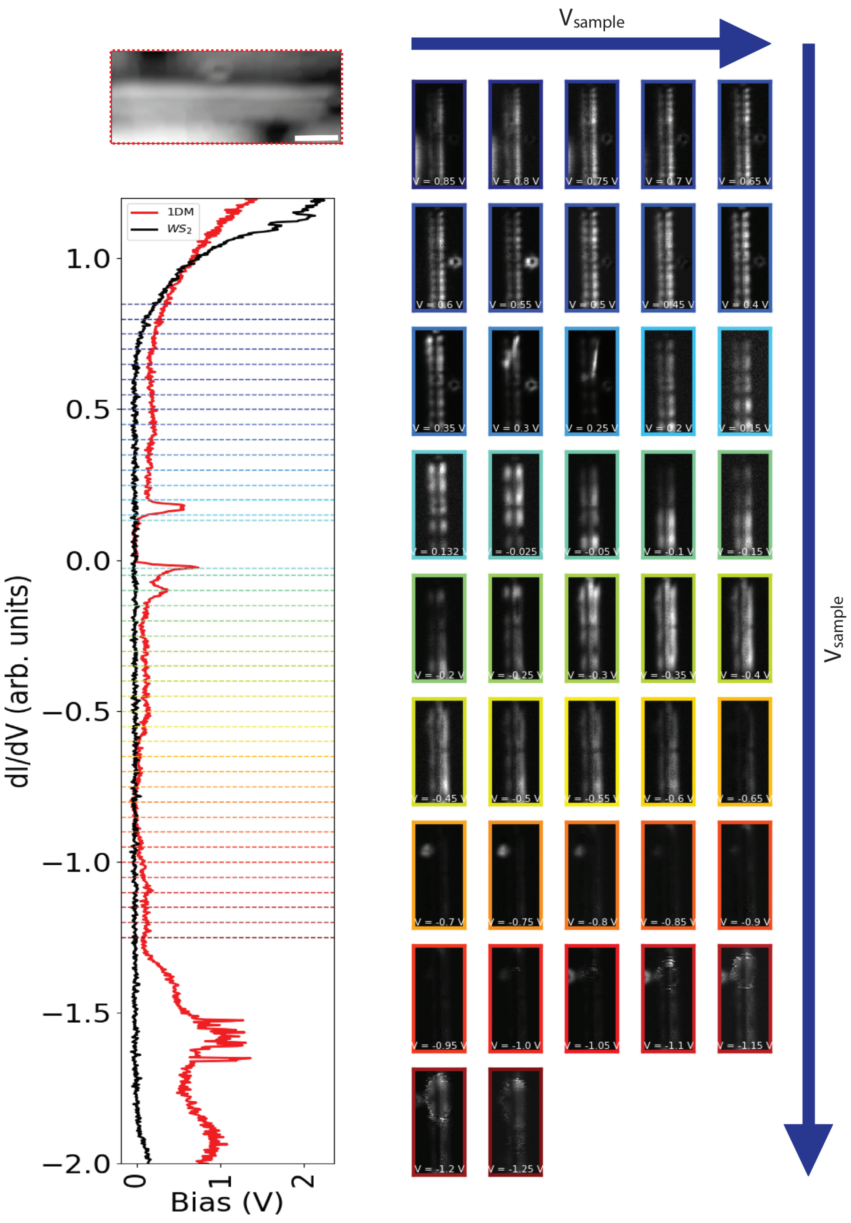

Supplementary Fig. 9: **Differential Conductance Mapping.** dI/dV mapping ($V_{modulation}$ = 5 mV) over the spectra region shown for a electron-like 1DM, with 4|4P intermediate structure, on a jet color scale, where the energy is ramped from near the VBM of WS_2 by 0.05 V to the HOS gap opening of the 1DM hosting a TLL, and then from the LUS to the CBM of WS_2. Arrows indicate decreasing bias. The defect imaged is shown to the upper left, where dI/dV images are representative of the region highlighted in dashed red (I_{tunnel} = 30 pA, V_{sample} = 1.2 V). Scale bar, 1.5 nm. A 1D particle in a box behavior is evident, and orbitals of both the TLL and a V_S can be visualized on as-acquired data at respective energies. Additionally, presence of a V_S scatters available quantum-well states (spatially-centered within the defect) above the HOS.

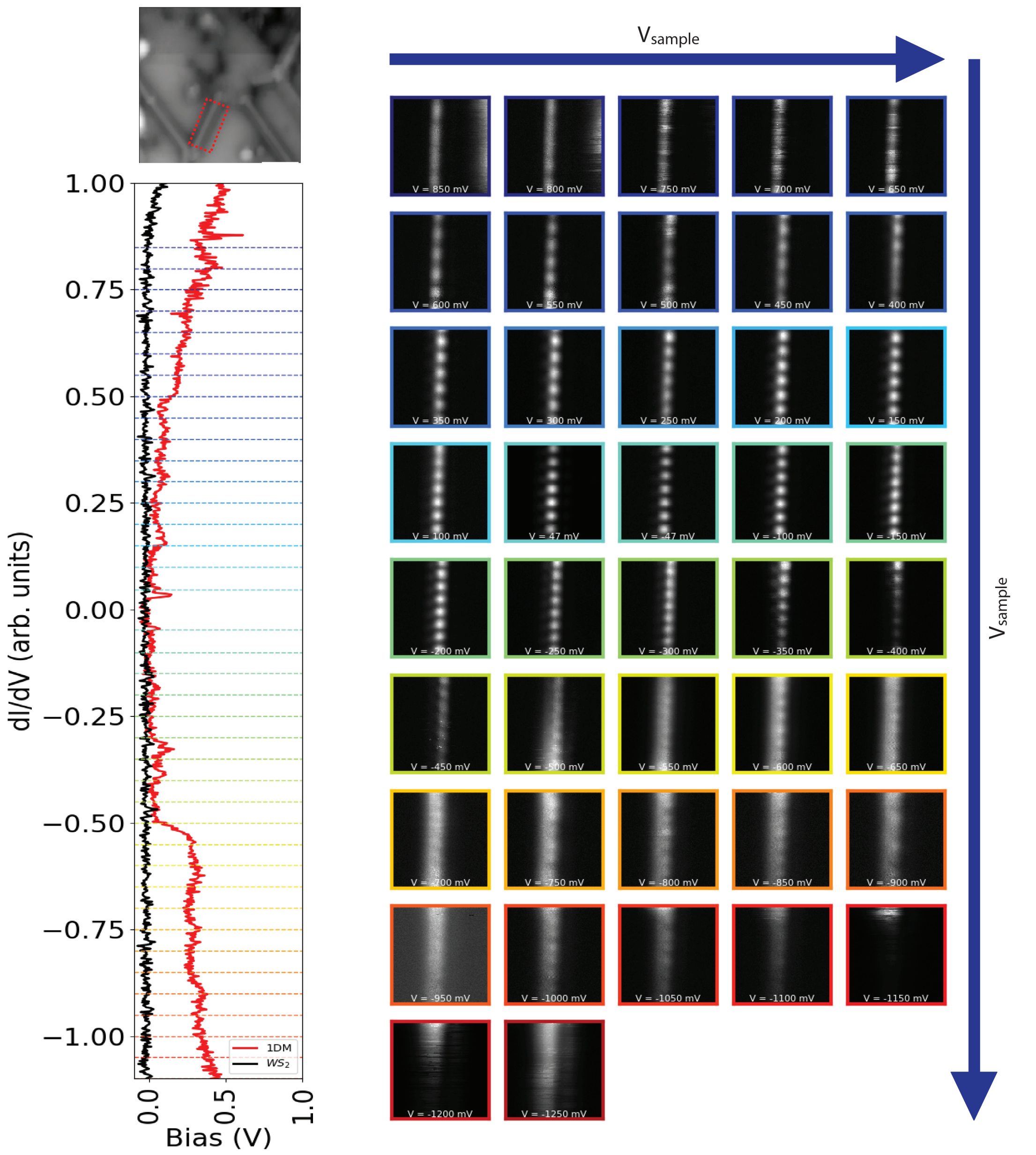

Supplementary Fig. 10: **Differential Conductance Mapping.** dI/dV mapping ($V_{modulation}$ = 5 mV) over the spectra region shown for a hole-like 1DM, with 4|4E intermediate structure, on a jet color scale, where the energy is ramped from near the VBM of WS_2 by 0.05 V to the HOS, and then from the LUS to the CBM of WS_2. The defect imaged is shown to the upper left, where the partial portion of the mapped defect is highlighted in dashed red (I_{tunnel} = 30 pA, V_{sample} = 1.2 V). Scale bar, 4 nm. Orbitals of the as-formed TLL can be visualized on as-acquired data as a function of bias voltage, where arrows indicate decreasing bias.

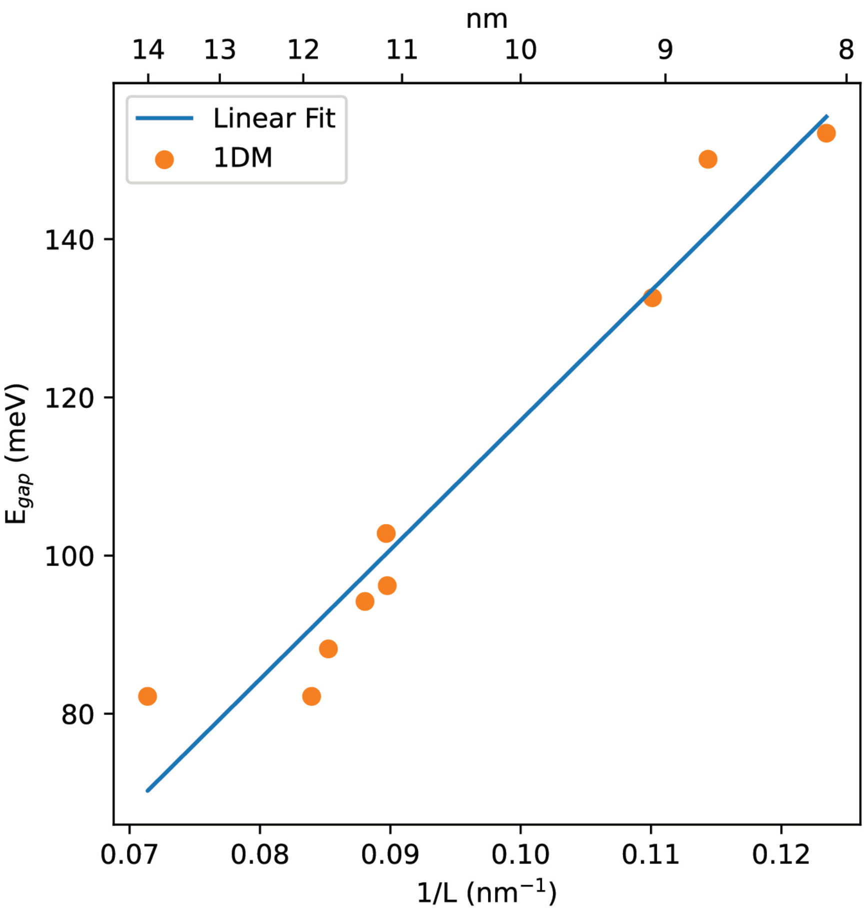

Supplementary Fig. 11: **Gap Length Dependence.** Band gaps are depicted across 9 1DMs, where each point represents the average of multiple reproducible data points, measured as a function of length (slope = 1636.4 ± 152.0 meV·nm, offset = -46.5 ± 14.7 meV). A linear relationship is shown across both 1DM structures. Fitting was performed using the lmfit package in Python[13].

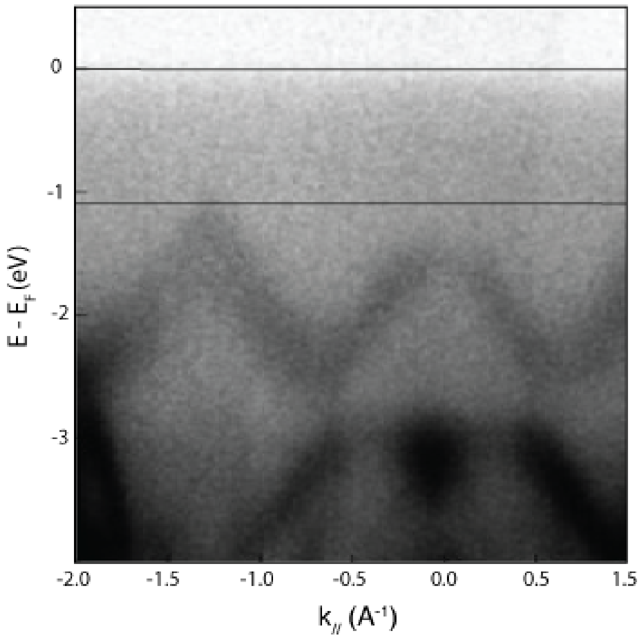

Supplementary Fig. 12: **nARPES Polarization Investigation.** WS_2 bands collected with linear vertical polarization. The horizontal line below E-E_F indicate the top of the valence band for the defective crystal.

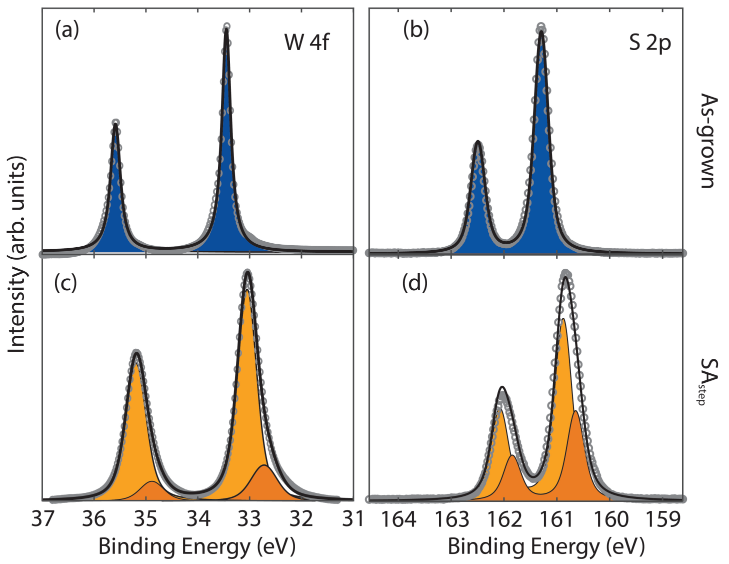

Supplementary Fig. 13: **Measured W and S Core Levels Spectra of As-grown and Defective WS_2.** (a) W $4f$ 7/2 and 5/2 levels centered at 33.5 eV and 35.7 eV, respectively, and (b) S $2p$ 3/2 and 1/2 core levels centered at 161.3 eV and 162.4 eV, respectively, from the unmodified sample. (c) and (d) are relative to WS_2 after SA_{step}, displaying two components (light and dark orange), with a relative shift of 0.4 eV for W $4f$ peaks and 0.2 eV for S $2p$ peaks.

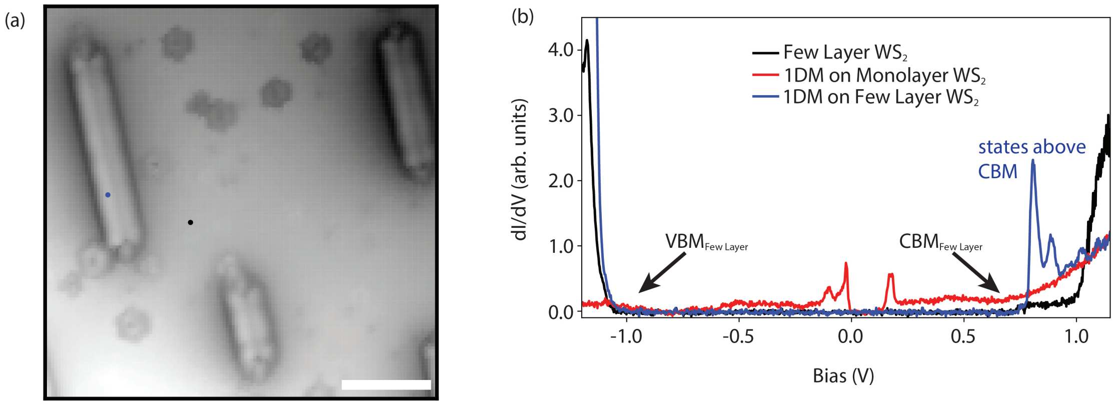

Supplementary Fig. 14: **LDOS on Multilayered WS_2.** (a) Scanning tunneling micrograph depicting 3 ML of WS_2 over MLG/SiC(0001) with both IDs and point defects (I_{tunnel} = 30 pA, V_{sample} = 1.2 V). Scale bar, 4 nm. (b) dI/dV point spectroscopy of 3 ML WS_2 (black), a 1DM within 1 ML WS_2 (red), and a 1DM within 3 ML WS_2 (blue). Corresponding spectral locations are depicted in (a), where both the VBM onset and CBM onset for the ID (blue) match that of the as-grown 3 ML WS_2 (black) spectra.

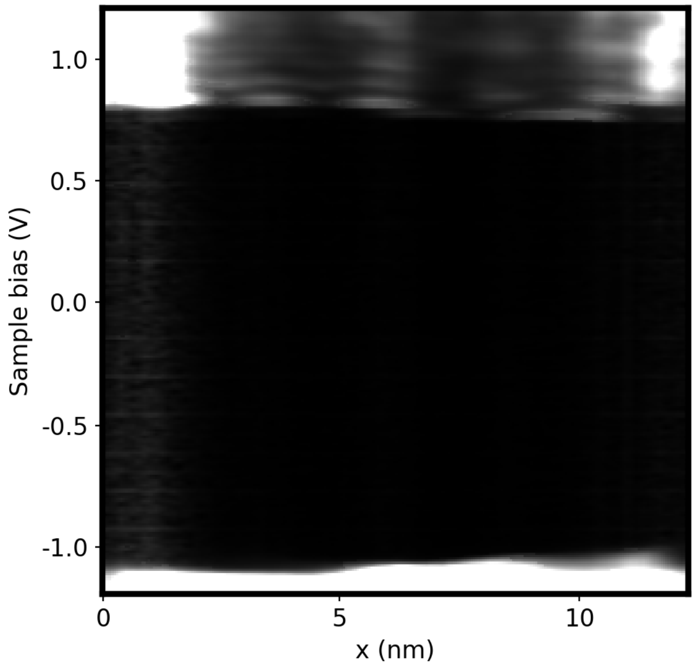

Supplementary Fig. 15: **Spatially Resolved Scanning Tunneling Spectroscopy over Multilayer WS_2.** Dense LDOS spectra (1x128x500 pixels) collected over an anticipated 1DM not in contact with underlying graphene ($V_{modulation}$ = 5 mV, I_{set} = 150 pA), where states within the gap are not present.

Supplementary References

[1] Ziegler, J. F., Ziegler, M., & Biersack, J. SRIM – The stopping and range of ions in matter. *Nucl. Instrum. Methods Phys. Res. B: Beam Interact. Mater. At.* **268**, 1818 (2010).

[2] Mitterreiter, E. et al. The role of chalcogen vacancies for atomic defect emission in MoS_2. *Nat. Commun.* **12**, 3822 (2021).

[3] Fox, D. et al. Helium ion microscopy of graphene: beam damage, image quality and edge contrast. *Nanotechnology* **24**, 335702 (2013).

[4] Komsa, H.-P. et al. Two-dimensional transition metal dichalcogenides under electron irradiation: Defect production and doping. *Phys. Rev. Lett.* **109**, 035503 (2012).

[5] Susi, T. et al. Isotope analysis in the transmission electron microscope. *Nat. Commun.* **7**, 13040 (2016).

[6] Chang, J., Cho, J.-Y., Gil, C.-S., & Lee, W.-J. A simple method to calculate the displacement damage cross section of silicon carbide. *Nucl. Eng. Technol.* **46**, 475 (2014).

[7] Haldane, F. D. M. 'Luttinger liquid theory' of one-dimensional quantum fluids. I. Properties of the Luttinger model and their extension to the general 1D interacting spinless Fermi gas. *J. Phys. C: Solid State Phys.* **14**, 2585 (1981).

[8] Fabrizio, M. & Gogolin, A. O. Interacting one-dimensional electron gas with open boundaries. *Phys. Rev. B* **51**, 17827 (1995).

[9] Anfuso, F. & Eggert, S. Luttinger liquid in a finite one-dimensional wire with box-like boundary conditions. *Phys. Rev. B* **68**, 241301 (2003).

[10] Kane, C., Balents, L. & Fisher, M. P. A. Coulomb interactions and mesoscopic effects in carbon nanotubes. *Phys. Rev. Lett.* **79**, 5086 (1997).

[11] Jolie, W. et al. Tomonaga-Luttinger liquid in a box: Electrons confined within MoS_2 mirror-twin boundaries. *Phys. Rev. X* **9**, 011055 (2019).

[12] Zhu, T. et al. Imaging gate-tunable Tomonaga-Luttinger liquids in 1H-$MoSe_2$ mirror twin boundaries. *Nat. Mater.* **21**, 748 (2022).

[13] Newville, M., Stensitzki, T., Allen, D. B., & Ingargiola, A. LMFIT: Non-Linear Least-Square Minimization and Curve-Fitting for Python. https://lmfit.github.io/lmfit-py/ (2014).
